# Health transitions in recently widowed older women: a mixed methods study

**DOI:** 10.1186/1472-6963-13-143

**Published:** 2013-04-18

**Authors:** Michelle DiGiacomo, Joanne Lewis, Marie T Nolan, Jane Phillips, Patricia M Davidson

**Affiliations:** 1 Centre for Cardiovascular and Chronic Care, Faculty of Health, University of Technology Sydney, PO Box 123, Broadway, NSW, 2007, Australia; 2 Department of Acute and Chronic Care, School of Nursing, Johns Hopkins University, North Wolfe Street, East Baltimore, MD, USA; 3 The Cunningham Centre for Palliative Care & The University of Notre Dame, Darlinghurst, NSW, Australia

**Keywords:** Widowhood, Older women, Health transitions, Health care utilization, Mixed method, Qualitative

## Abstract

**Background:**

Older recently widowed women are faced with increased health risks and chronic conditions associated not only with bereavement, but also, older age. Loss and grief, adjusting to living alone, decreased income, and managing multiple chronic conditions can impact on older women’s ability to transition following recent spousal bereavement. Providing appropriate, timely, and effective services to foster this life transition is of critical importance, yet few services directed towards these women exist in Australia, and there is little data describing the experiences of women and their support needs at this time.

**Methods:**

We conducted a longitudinal mixed method study using in-depth semi-structured interviews and questionnaires that were administered three times over a twelve month period to understand the experiences and needs of older women in the period following their husbands’ deaths. Descriptive statistics and Interpretive Phenomenological Analysis were used to analyse quantitative and qualitative data, respectively, prior to data integration.

**Results:**

Participants were twenty-one community-dwelling recently widowed older women who were an average age of 71 (SD 6.13) years. The majority of participants scored within normal ranges of depression, anxiety, and stress, yet a subset of participants had elevated levels of each of these constructs (37%, 27%, and 19%, respectively) throughout the study period. Positive self-reports of general health predominated, yet 86% of participants were living with one or more chronic condition and taking an average of 4 medications per day. The majority (76%) experienced exacerbations of existing conditions or were diagnosed with a new illness in the early bereavement period, leading to planned and unplanned hospitalisations and other health service use. Qualitative data provided insight into these experiences, the meanings women ascribed to them, and their reasons for enacting certain health risk behaviours.

**Conclusions:**

The combination of co-morbidities, polypharmacy, and risk behaviors impacted on medication management and appeared associated with health events. The feminization of ageing and an increasing number of older women living alone with multiple chronic conditions represent significant challenges to health services and societal support systems. Older women’s transition to widowhood signals concomitant health transitions and multidimensional support needs.

## Background

Older women are increasingly represented in today’s society and are particularly at risk for a range of chronic health conditions and economic deprivation [1]. A recent article by Richmond identifies that among centenarians, the fastest growing age segment in the Australian population, 75% will be women [2]. Widowhood is an important, yet common life event that requires a significant amount of adjustment. Despite literature emphasising the eventual resilience of women [3], where they generally adjust well and continue to live fulfilling lives, there is evidence that the early bereavement period (the first 2 years following death of the husband) carries with is several risks to health, social, and economic wellbeing [4].

A review of health outcomes of recently bereaved people found early increased health risks including increased hospitalisation, medication use, changed eating habits, living arrangements, and social relationships [5]. Moreover, recently widowed older women have increased rates of hospitalisation [6]. Women are faced with increased health risks and chronic conditions associated not only with bereavement, but also, middle and older age. Older women have higher rates of severe disability and this continues as they age. Increases in depression, anxiety, and loneliness [7,8] have also been reported. Many older women live alone upon spousal bereavement [9]; an arrangement that may impact on their daily routines, ability to self-manage chronic conditions, as well as economic resources [10]. These women often have a decreased income upon spousal death, particularly if they had not been in paid employment for long and have no or little retirement savings. They are more likely to live in poverty than men or their married counterparts, and may suffer from financial and housing insecurity and reduced income despite maintained or increased expenses [11,12].

These factors mean that older women may be less equipped to address the challenges of widowhood. Poor physical and psychological health outcomes highlight the need for health professionals and services to be aware of and responsive to issues faced by older people who have recently lost a spouse [8]. Providing appropriate, timely and effective services to foster this life transition is of critical importance, yet few services directed towards these women exist in Australia, in contrast to the number of services offered to assist and support caregivers.

Australian society faces major challenges as women age and have fewer options for support. Improving current services or implementing new and innovative programs to better address the needs of widows can decrease not only their economic burden, but will decrease the economic costs incurred by the health system associated with hospitalization rates and inabilities to self-manage. Although there have been many studies on widows’ health outcomes, there is little data describing the experiences of women and their support needs at this time. To address this dearth and to inform design and delivery of services, we undertook a study on recently widowed older women. This paper provides a snapshot of health and health service use during the early widowhood period of older women.

## Methods

This study used a longitudinal mixed methods design using qualitative and quantitative methods to describe experiences of older recently widowed women. Each woman participated in in-depth semi-structured interviews and completed questionnaires at three time points over a 12 month period (baseline/Time 1, Time 2 (6 months after Time 1), and Time 3 (6 months after Time 2). Using two methods concurrently augmented detail, gave meaning, and added depth to the experiences described by participants. The longitudinal design allowed observation of behaviours along the early trajectory of widowhood.

### Recruitment

Participants were recruited through study advertisements distributed as part of local and national health and social service organization newsletters, organization membership lists, or via websites targeting older women and/or health professionals. Study brochures were available at two palliative care memorial services. Participants were included if they were women whose husband/partner died within the previous two years and were aged 65 or older. Individuals were excluded if they did not speak English or were not able to participate in interviews. Potential participants or individuals they nominated, such as a family member or general practitioner (GP), contacted the researcher to discuss participation. Informed consent documentation was mailed to participants with the first set of questionnaires. Upon receipt of these completed documents, the researcher scheduled interviews with participants.

### Instruments

Demographic, service utilization, health and medication information was collected using investigator-developed questionnaires. Demographic information included age, socioeconomic characteristics, living arrangements, years married, and number of children. Service utilization included the number of times a participant accessed different health professionals or services over the previous 6 months. Participants were also asked to indicate current diagnoses and medications.

Only at Time 1, risk of complicated bereavement was assessed by the interviewer using a modified version of Parkes’ Bereavement Risk Index-Modified (BRI-4) [13,14]. This instrument contains 4-items that assess the bereaved person’s emotional responses and coping abilities. It has demonstrated acceptable reliability and validity and good predictive validity when correlated with outcome measures at three months following the death.

At all three data collection points, participants completed these questionnaires on service utilization and health conditions and medication use. In addition, they completed the Short-Form 12-Item Health Survey (SF-12v1) and the Depression, Anxiety and Stress Scales (Short Form) (DASS 21). The SF-12v1 is a self-administered, closed-ended, multi-purpose, health survey that assesses several domains of health-related quality of life over the past 4 weeks. The twelve items assess physical functioning, role limitations, bodily pain, health perception, energy level, social functioning, and psychological well-being, resulting in physical (PCS) and mental composite scores (MCS). The SF-12 has undergone extensive validation (test-retest reliability for PCS = 0.89; MCS =0.76) and demonstrated good construct validity [15,16].

The DASS 21 contains three self-report scales that measure the negative emotional states of depression, anxiety, and stress experienced over the past week. Respondents indicate the extent to which each statement applied to them from ‘not at all’ to ‘applied to me very much or most of the time’ [17]. Ratings are summed and multiplied by 2 to arrive at a severity rating for each construct, from normal to extremely severe. Alpha values based on a normative sample for the 7-item scales are: depression 0.81; anxiety 0.73; stress 0.81 [17]. This scale has been used in Australians following cardiac events and demonstrated reliability and the ability to document changes over time [17].

### Interviews

In addition to completing questionnaires at three time-points, each participant engaged in in-depth semi-structured interviews three times within a twelve month period. Interviews lasted from 1–3 hours and took place over the phone or at participants’ homes. The female researcher was experienced in qualitative methods and working with older women in the context of managing chronic conditions. The interview schedule reflected components of the ecological framework and life course and role theories guiding this study, yet discussion was not limited to these topics. A priori topics included the experience of the husband’s death, social support, relationships, living arrangements, health status, health behaviors, and perceived met and unmet needs for information or support. Contextual information regarding responses to questionnaires was also elicited. Time 2 and 3 interviews centred on how participants had been over the previous few months, re-visiting some earlier topics, and clarifying and validating the researcher’s interpretations of previous interview data. Recent events or changed circumstances were discussed.

### Analysis

Interviews were audio recorded with permission of participants and transcribed. The Interpretive Phenomenological Analysis (IPA) of transcripts [18] involved reading the transcripts repeatedly while taking notes in margins and developing a coding framework. IPA is a systematic process commonly used in psychological research and it stems from phenomenological and interpretive traditions. Thus, the researcher’s critical perspective was ‘bracketed’ to facilitate concentration on the participants’ experiences and meaning-making. A reflexive diary was used to record the researcher’s critical comments. Themes were identified following categorization of codes and then examined for connections. Concepts were clustered to develop an analytical framework as analysis proceeded with new themes re-assessed and revised as necessary. Quantitative data was entered into SPSS for descriptive analysis. Qualitative and quantitative data was then integrated.

IPA is a set of systematic processes that shift from phenomenological to interpretive while maintaining a commitment to understanding the participant’s point of view and a psychological focus on personal meaning-making in specific contexts [18]. This approach is iterative, inductive, and flexible.

### Ethical considerations

Approval to conduct this study was granted by the affiliated Hospital and University human research ethics committees. Participants were contacted on the day following interviews to ensure they were not distressed. If requested, perceived necessary, or indicated on DASS 21, the researcher facilitated links with relevant support.

## Results

Twenty-four women were recruited, although three were unable to participate in the initial interviews and were thus excluded. Reasons were inability to allocate time for the interview due to multiple competing demands, in one case because one woman’s home had been flooded soon after agreeing to participate. One of the included participant’s health decline and subsequent death meant she only completed the first interview and Times 1 and 2 questionnaires. In total, the data were comprised of 61 interviews and 83 questionnaires across twenty-one participants who were an average age of 71 years. Although participants each had an average of 2 children, five women had no children (Table 1). They had mainly been married to their late husbands for decades until his death an average of one year prior to the study. The majority of participants had been caregiving for their husbands who were unwell prior to death.

**Table 1:** Participant characteristics (N = 21)

**Characteristic**	**Mean (SD)**	**Range**
Age	71.43 (6.13)	63 - 82
Number of children	2.24 (1.58)	0 - 5
Number of years married to spouse who recently died	43.14 (15.12)	8 - 63
At 1st interview, number of months since death of husband	14.29 (11.07)	2 - 47
At 2nd interview, number of months since death (n = 20)	20.14 (11.10)	8 - 53
At 3rd interview, number of months since death (n = 20)	25.8 (11.16)	13 - 59
	**%**	**N**
Carer for husband at time of death	81	17
Living alone	95	20

### Bereavement risk index-modified (BRI-4)

The Bereavement Risk Index-Modified (BRI-4) provided an assessment of participants’ emotional wellbeing at the first data collection interview only, as perceived by the interviewer. Scores recorded by the interviewer indicated that the majority of participants were considered to be at low risk of experiencing complicated bereavement, yet a subset were at medium (28.6%, n = 6) and high risk (4.8%, n = 1). Perceived anger and self-blame were the most common contributors to the interviewer’s risk perception. In the few cases where this was evident, these feelings generally reflected participants’ perceptions of inadequate care provided by health professionals, health care organisations, or the health care system.

### Depression, anxiety and stress scales (DASS-21)

**Figure 1:**
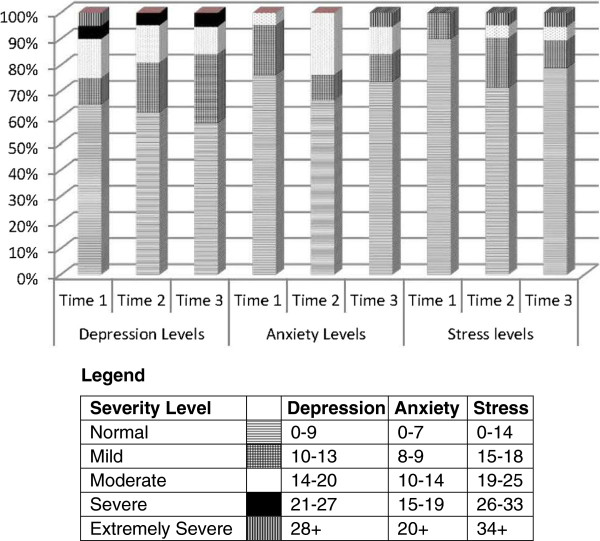
DASS21 levels of severity by data collection period (N = 21; *N = 20).

**Figure 2:**
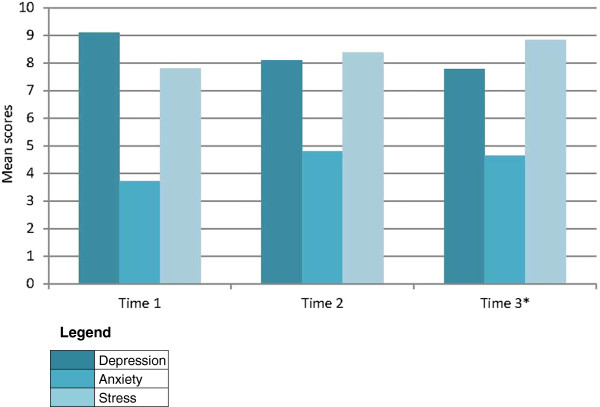
DASS-21 mean scores by data collection period (N = 21; *N = 20).

The majority of women scored within normal ranges of depression, anxiety, and stress, yet a subset of participants had elevated levels of each of these constructs (Figure 1). Only mild levels of depression increased from Time 1 to Time 3, whereas all other levels were maintained or declined, with the largest decline in the normal level or no depression. In fact, on all three subscales, normal levels declined somewhat over the 12 month period. Mean scores for each construct over the 12 month data collection period are depicted in Figure 2. Peaks in anxiety and stress levels appeared at time 2.

Although the DASS-21 stress subscale depicted a small proportion of women who were experiencing higher than normal stress levels, participants’ descriptions of their stress levels following the death of their husband, even months later, often indicated more pronounced strain and hassle. Stressors involved administrative, financial, and health burdens. Such contrasting findings may reflect item validity, participants’ interpretations of questionnaire items, denial or fluctuation of emotion, or recall facilitated by interviews. The following excerpts depict two participants’ psychological states in early bereavement:

I lost a lot of energy; I got into a lot of ‘couldn’t be bothered’. I felt overwhelmed. I get overwhelmed easy. Iris

I am overweight at the moment and that’s because I’ve been very non-active. Usually I go walking on the beach or something like that but for the last 6 months I haven’t. I’ve just been, as I say, sort of holed up at home. Lydia

### Short-form 12-item health survey (SF-12v1)

**Figure 3:**
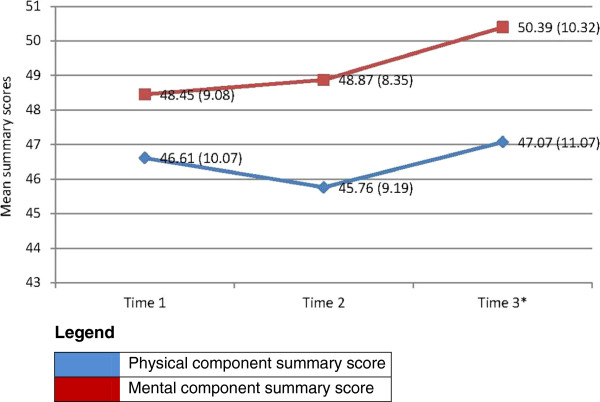
SF-12 physical and mental component summary means (SD) (N = 21; *N = 20).

SF-12v1 scores indicated that participants generally rated their health favorably and at no time did any participant describe their health as poor. Mean scores on the physical health composite score (PCS) decreased at time two and rebounded at Time 3 (Figure 3). Mean mental health composite scores (MCS) were higher than PCS and increased moderately, yet steadily throughout the study period. These changes indicate that participants were, on average, reporting more favorable physical and mental wellbeing at Time 3 than they did at Time 1.

Despite the predominantly positive self-reports of health in general, most of the women were living with at least one (86%) or two or more (67%) chronic conditions that impacted on their daily functioning. Descriptions of conditions, symptoms, and ways in which these impacted on participants were expounded during interviews. Reports of living with pain, self-management activities, and difficulty with activities of daily living dominated:

I’ve got a hole in my leg that goes right down to a stainless-steel knee joint. Golden Staph. That’s what’s wrong with my leg. This is my second knee replacement…Around the twelve month mark, I go in, they open it all up, wash and clean it all out, and close it back up again and leave the hole there so as it can drain. I’m left to do the dressings on my own each week. Alice

This woman’s daily life is significantly affected by this and other conditions, including diabetes. She described mobility impairment, ongoing costs associated with regular dressings, and pain requiring medication and frequent doctor visits. Others noted routine activities such as food shopping and house cleaning were challenged by pain. Although many of these chronic conditions pre-existed their husband’s death, participants experienced exacerbations and were diagnosed with new conditions in the early bereavement period.

### Health events

Health events, defined as newly diagnosed conditions, exacerbation of existing conditions, or incidents requiring health professional intervention, occurred in the period following the husband’s death (Table 2). These events were mostly captured by the health service utilization instrument, although omissions were recalled during interviews. These events ranged in magnitude and impact and were widely experienced with 76% of participants reporting at least one event during the study. On average, almost 1 out of every 2 participants (45%) reported at least one event every six months over the study period that impacted on their ability to access health care and self-manage. Below is an example of the impact a health event had on one woman’s daily life:

That (shingles) happened about 2 to 3 months afterwards (husband’s death)…So that was a sure sign that things weren’t right…. It stopped me from doing a lot of things because I had to do them with funny vision, but I was able to do what I had to do and got through it. But looking back, it wasn’t very nice. I have to be busy and creative to be at my happiest. Emma

**Table 2:** Health events, service access, and medication use (N = 21; *N = 20)

	**Time 1**	**Time 2**	Time 3*
# who reported health events	10 (48%)	8 (38%)	9 (45%)
# of events	18	9	20
*Falls*	1	1	3
*Medication-related*	1	0	1
*Exacerbation/new illness*	14	5	9
*Surgery*	3	2	7
Emergency Department presentation	4	3	3
Hospital admission	6	1	5
Planned hospital admission	4	5	5
Unplanned hospital admission	4	2	5
**Health professional/service access**	**Mean # times accessed (SD)**		
Accessed GP	3.33 (2.55)	3.57 (4.03)	2.84 (2.01)
Accessed Practice Nurse	0.24 (0.89)	0.38 (0.81)	0.63 (1.54)
Accessed Pharmacist	3.00 (4.44)	2.57 (3.80)	2.00 (2.65)
Accessed Physio	0.38 (1.36)	0.57 (1.47)	0.42 (1.31)
Accessed Massage	0.10 (0.30)	0.29 (1.10)	0.05 (0.23)
Accessed Chiropractor	1.57 (5.51)	0.95 (2.42)	1.05 (2.53)
Accessed Naturopath	0.00 (0.00)	0.29 (1.10)	0.00 (0.00)
Accessed Psychologist/Psychiatrist	0.62 (1.53)	1.00 (2.85)	0.68 (1.89)
Accessed Support Group	1.10 (3.49)	1.29 (4.10)	1.26 (3.78)
Accessed Bereavement	0.05 (0.22)	0.05 (0.22)	0.32 (1.38)
Accessed Outpatient Clinic	0.05 (0.22)	0.10 (0.44)	0.16 (0.38)
Accessed Home Care	0.05 (0.22)	0.02 (0.11)	0.37 (1.38)
Accessed Meals on Wheels	0.00 (0.00)	0.00 (0.00)	0.05 (0.23)
Accessed Nursing	0.05 (0.22)	0.05 (0.22)	0.11 (0.46)
**Medication Use**		**% Participants (N)**	
Hypertension	63.2 (12)	71.4 (15)	60 (12)
Blood Thinner	21.1 (4)	38.1 (8)	30 (6)
Cholesterol	36.8 (7)	38.1 (8)	30 (6)
Diabetes	15.8 (3)	28.6 (6)	20 (4)
Anti-uricemic	0 (0)	4.8 (1)	5 (1)
Diuretic	15.8 (3)	14.3 (3)	5 (1)
Analgesic	21.1 (4)	9.5 (2)	15 (3)
Anti-inflammatory	21.1 (4)	4.8 (1)	5 (1)
Osteo	10.5 (2)	14.3 (3)	5 (1)
Hormone/Steroid	10 (2)	14.3 (3)	15 (3)
Antidepressant	21.1 (4)	23.8 (5)	15 (3)
Insomnia	21.1 (4)	4.8 (1)	5 (1)
Gastrointestinal	31.6 (6)	33.3 (7)	35 (7)
Penicillin	5.3 (1)	9.5 (2)	0 (0)
Antibiotic	9.5 (2)	9.5 (2)	5 (1)
Respiratory	15.8 (3)	19 (4)	10 (2)
Allergy	5.3 (1)	4.8 (1)	10 (2)
Vitamin supplement	57.9 (11)	52.4 (11)	45 (9)
Herbal	28.6 (6)	42.9 (9)	20 (4)

For this woman and others, these were not isolated events, but often began a cascade of episodes that challenged their wellbeing. At Time 2, a woman reported fracturing her scapula from a fall, inhibiting her self-care capacity for months. At Time 3, she reported having had an episode of breathlessness and feeling ‘waterlogged’ which she associated with not readily refilling her blood pressure medication. She attributed this to her distressing financial and living situation. Upon advice received over the telephone from her health professional daughter, she phoned an ambulance, was admitted to intensive care for five days, diagnosed with heart failure, and prescribed warfarin. Each of these events had serious implications for her ability to self-manage, particularly while living alone, in precarious housing, with nearest family located hours away.

Participants commented that being sick while living alone was an anxiety-provoking experience. They feared a recurrence with no one to assist them:

…very frightening being on your own when you’re ill. Gloria

If something happens during the night, there is no one to call; I wouldn’t call a neighbor in middle of night. Debra

Examples of other major health events included acute urinary retention, complete transient heart block, and fractured lumbar discs resulting from a fall. Fear and anxiety were common to participants’ experiences because they were at home alone, in pain and immobilized, and unsure of what was happening. Participants mainly accessed emergency services in these cases. One woman’s pain prevented her from driving herself to her doctor, so she endured an excruciating bus trip with her overnight bag, confident she would be admitted to hospital once visiting her doctor. Her insistence on being admitted was informed by pain, as well as her lack of transport and home care assistance.

### Health care utilization

Participants reported whether and how often they accessed different types of health professionals or health/social services in the previous six months. Several emergency department presentations (14-19% of participants) and hospital admissions (5-29% of participants) occurred throughout the study period. Most hospital admissions were planned, but many were unplanned, indicating that they arose from a recent health event or exacerbation of a condition (Table 2).

Participants reported the number of visits they made to a variety of health professionals throughout the study period (Table 2). The most accessed health professionals at Time 1 were GPs, pharmacists, and chiropractors. Support group access and visits to mental health professional peaked at Time 2, potentially representing a period in which participants were experiencing increased need for this type of support or increased awareness of this support. GPs and pharmacists were most frequented at each time point.

Relationships with GPs varied. Several participants were long-time patients and described their GP as a critical contact and support person. Regardless of relationship duration, some participants explained that they would not feel comfortable discussing certain matters with their GP, such as mental health issues, including feelings of depression. One woman avoided her GP because she expected she would become emotional in his presence. Another woman had shown signs of depression during the interview and when asked if she had talked to her GP about this, she stated that this was not something she would talk about with him.

Few women accessed formal bereavement services at Time 1, preferring instead to manage their grief and other circumstances alone. Four women had been offered bereavement support by their GP, but refused. Rather than being perceived as something that could aide coping, one woman commented that counselling would be one additional thing to cope with:

I knew I could go there (counselling) if I needed to, but I kept sort of waiting until I felt I could cope with the counselling and I was doing other things… I think there was a feeling that if you started to talk about those sorts of things you’d lose control. Beth

Some perceived this support to be for people who had more severe difficulties than they were experiencing and others explained that they had not been offered this type of support. Upon conclusion of the study, almost a third of participants had accessed either individualized or group bereavement services at least once. The following is one participant’s perception of beneficial involvement in a bereavement support group:

It permits you to talk about your husband and his death and dying and I know it sounds morbid, but I think it helps to get it out. I can talk about it over and over and I think it does me good. Rebecca

### Health behaviors: medication adherence, alcohol consumption, and eating

The mean number of medications taken was 4 (prescription and over-the-counter). The majority of participants (60-71%) reported taking hypertension medication throughout the study followed by vitamin and mineral supplements (45-58%) (Table 2). Reports of cardiovascular disease-related medications were somewhat more frequent at Time 2 than other data collection periods. Fluctuations in reporting may reflect inconsistencies of self-reporting or they may represent biological changes affected by this period of grief. One participant who had previously managed her warfarin well, reported difficulties managing in the months following her husband’s death when she was unable to sleep and lost weight.

Medications reported more at Time 1 than other times were analgesics, anti-inflammatories, sleep aides, diuretics, and supplements. Sleep aides were prescribed by GPs, particularly in the period immediately following their husband’s death. Sleep dysfunction persisted for several participants:

I hate night-time. In the daytime I am busy, but at night time I hate it. I’m not frightened though. Just getting into bed by myself at night time is what I hate…Monday night was a terrible night. I was sort of up all night. My mind just doesn’t stop, you know? Carol

Participants who used sleep aides at Time 1 reported having ceased or reduced use at Times 2 and 3, maintaining their prescriptions, yet only using periodically, as needed. Although struggling with insomnia, several participants were opposed to using sleeping tablets:

My major problem is that I can’t sleep. My GP wanted to give me pills and I said, look I don’t really want to go down that path. Because I’m on my own, if I don’t sleep I’m not disturbing anyone. If I want to sit up and read until 4 o’clock or listen to the radio or something like that, I don’t get up until 11 o’clock because I haven’t gone to sleep until say 5 o’clock. I’m not really hurting anyone. But some days I’m just exhausted and I’ve been putting off taking medication for the lack of sleep. I feel that after all this time I would have settled down, but I haven’t. Lydia

Participants’ discussed their reluctance to use sleep aides as related to their general orientation to medication-taking and perceptions that they were habit-forming. Perspectives of anti-depressants were likewise varied. Circumstances surrounding the death of their husband and peripheral stress contributed to some participants’ grief. One participant discussed her reticence to succumb to antidepressants despite her GP’s endorsement:

All my doctor wanted to do was give me antidepressants. She tried to convince me that I was depressed and I said I am not depressed. ‘I am tired out, I am lonely, I am sad, but I am not depressed,’ and I refused to have antidepressants until October when I was just desperate for sleep. I said, look, I’ve just gotta get some sleep. And she said ‘I’ve got some sleeping pills, but they’re an anti-depressant.’ Fran

Another woman explained that she had been taking anti-depressants prescribed by her GP for nearly four years without review, but had never visited a psychologist, nor had that been suggested. One woman reported that she was taking ‘*about eleven different tablets*’, although she did not know what they were all for because her husband used to organize her tablets for her:

The doctor put me on some tablets. They’re for reflux and to stop ulcers and that. Well, I took them for a while and I stopped them, but I’ve been on them again over the last five months, but I’ve stopped them again now because I didn’t want to get too used to them. Debra

The loss of her husband who contributed to her medication management resulted in her inconsistent self-management. From these discussions, it appeared that women did not always understand why they were taking some medications and were not always taking these medications as prescribed for therapeutic benefit. Medication adherence was variable and reflected participants’ beliefs and understandings or lack of understanding. Reports of off-label use included one participant taking antidepressants ‘*only on occasion*’ and ‘*with a couple glasses of wine*’ to help her sleep or improve her mood. Another participant who had been prescribed statins to manage her cholesterol ceased use after one month without consultation with her physician. She believed that incorporating more physical activity would manage her cholesterol satisfactorily; although her avoidance of her doctor signaled awareness that this was not advisable behavior.

### Food and alcohol consumption

As alluded to previously, some participants discussed using alcohol to combat insomnia and that their consumption had changed since their husband’s death:

Rebecca: I believe I drink too much, but I don’t get drunk. Maybe 4 or 5 glasses each day, starting at lunch time. Probably too much.

Interviewer: So is this different for you?

Rebecca: I think I’ve probably drunk a bit more since he’s died than beforehand…In the last six months, I suppose, when I’ve become more reclusive, I have increased my alcohol content and I think that gets me to sleep but it doesn’t keep me asleep. On the days that I don’t have any alcohol, I’m just up all night.

Decreased appetite and lack of motivation to prepare food sometimes meant participants consumed convenient nutritionally unsound food that required minimal preparation. Barriers to cooking were loneliness associated with cooking and eating alone and difficulty transitioning to preparing single portions. After years of cooking for her husband and children, loss of this caregiving role made the act of food preparation seem futile:

It (cooking) just seems pointless…Now that I don’t have to cook, I don’t do much. Eating is scrappy. I got through last year on chicken soup. Iris

In some cases, changes to eating behaviors resulted in unintentional weight loss. Although not perceived as a negative outcome for some participants, weight loss impacted on certain medications, such as warfarin.

## Discussion

Women’s high rate of chronic conditions and health events in this period following their husband’s death signals their increased needs and use of health care services, support, and transport, particularly hospitalization, specialists, and ambulance services. Although a subset of women experienced extended periods of depression and anxiety, anxiety around health events was prominent, particularly while living alone. Although we have focused on a widely recognized stressful life event in a population with unique issues, our findings reflect those of Longman et al. [19] who identified co-morbidities, health behaviors, and living arrangements as associated with older people’s frequent hospitalizations.

There were mild improvements on scores of physical and mental functioning over the study period. MCS was consistent with the mainly normal DASS-21 levels, however it was lower than means reported for US (United States of America) women aged 65 and over and Australian women aged 70–74 [20]. Presumably, grief related to recent spousal bereavement can likely account for these discrepancies. Although PCS means were within the US and Australian population norms, physical and mental health appeared to inhibit engagement in activity. Thus, it seemed that health mediated adjustment to widowhood by impeding re-engagement.

GP and pharmacists were most often patronized throughout this period, indicating they may be important channels of information, particularly regarding medication use and other health behaviors. Discomfort in disclosing emotional or psychological distress highlights the importance of availability of other avenues to receive needed support.

Changes to health behaviours both resulted from conditions and contributed to health conditions or their impact. Misunderstandings or a lack of knowledge regarding reasons for taking certain medications, fears of addiction, and perceptions of efficacy and need mediated adherence to medications. Grief resulted in engagement in risk behaviors such as self-medication with alcohol and medication variations. The combination of co-morbidities, polypharmacy, and risk behaviors impacted on medication management and was associated with health events. Sleep problems were pervasive and enduring and led to sometimes dangerous management tactics. Sleep difficulties have previously been identified as a serious problem in older women [21]. Our finding that a proportion of the sample used prescription sleep aides is consistent with previous research [22]. Our findings further suggest that usage declined throughout the study period. Fluctuations in medication-taking may reflect inaccurate self-reporting or may point to biological changes related to psychological demands within the context of recent spousal bereavement – a combination linked to major health events [23]. These findings signal need for awareness among women, health professionals, and support people regarding the potential impact of grief and related behaviors on medications.

### Strengths and limitations

The small sample size precludes capacity to undertake meaningful statistical tests and extrapolate to larger populations. Recruitment of participants at different points within the early bereavement period hindered conclusions regarding changes over time. Errors in self-report may have biased findings, although repeated interviews provided opportunities to correct and contextualize reports. The mixed method longitudinal design facilitated collection of contemporaneous and contextual data useful in service planning. Interviews served a dual purpose of facilitating in-depth descriptions of participant experiences and meanings as well as assessing questionnaire feasibility and acceptability.

## Conclusions

The feminization of ageing and an increasing number of older women living alone with multiple chronic conditions represent significant challenges to health services and societal support systems. Older women’s transition to widowhood signals concomitant health transitions and multidimensional support needs. Ongoing health events and related anxiety indicate potential for increased social isolation, challenges to self-management, and increased need for support. Increased awareness of issues associated with recent spousal bereavement in older women and innovative strategies are required.

## Abbreviations

GP: General Practitioner; IPA: Interpretive Phenomenological Analysis; BRI-4: Bereavement Risk Index-Modified; DASS-12: Depression, anxiety and stress scales (short form); SF-12v1: Short-Form 12-Item Health Survey; MCS: Mental Component Summary; PCS: Physical Component Summary; US: United States of America.

## Competing interests

The authors declare that they have no competing interests.

## Authors’ contributions

MD contributed to study design, data collection and analysis, and manuscript drafting. JL contributed to data analysis and revising the manuscript. MN contributed to planning the study and revising the manuscript. JP contributed to planning the study and revising the manuscript. PMD contributed to designing the study, data analysis, and revising the manuscript. All authors read and approved the final manuscript.

## Pre-publication history

The pre-publication history for this paper can be accessed here:

http://www.biomedcentral.com/1472-6963/13/143/prepub
